# Modern contraceptive use among women of reproductive age in Ghana: analysis of the 2003–2014 Ghana Demographic and Health Surveys

**DOI:** 10.1186/s12905-018-0634-9

**Published:** 2018-08-20

**Authors:** Philomina Akadity Aviisah, Samuel Dery, Benedicta Kafui Atsu, Alfred Yawson, Refah M. Alotaibi, Hoda Ragab Rezk, Chris Guure

**Affiliations:** 1 Department of Biostatistics, School of Public Health, College of Health Sciences, University of Ghana, Legon-Accra, Ghana; 2 Department of Population Family and Reproductive Health, School of Public Health, College of Health Sciences, University of Ghana, Legon-Accra, Ghana; 3 Department of Mathematical Sciences, Faculty of Science, Princess Nourah bint Abdulrahman University, Riyadh, Saudi Arabia; 4 Faculty of Commerce AL-Azhar University (Girls’ Branch), Cairo, Egypt; 5 College of Health and Well-Being, Department of Health Information Management, Kintampo, Ghana

**Keywords:** Modern contraceptive, Use of contraceptives, Women, Reproductive age, Ghana

## Abstract

**Background:**

Contraceptives are used in family planning to space or limit pregnancies and are categorized into modern and traditional methods. The modern methods have been proven to be more scientifically effective at preventing unwanted pregnancies than the traditional methods. With data from three (3)-different Demographic and Health Surveys, the aim of this study is to assess the trends and identify factors that consistently influence modern contraceptives’ use among women of the reproductive age group in Ghana.

**Methods:**

The study used secondary data from the 2003, 2008, and 2014 Ghana Demographic Health Surveys (GDHS). The trends of determinants of modern contraceptives use among women of reproductive age in Ghana were determined. A bivariate approach was used to select significant predictors. The Cox proportional hazards model analysis was employed via a multilevel modelling approach.

**Results:**

Out of the total respondents of 2229, 2356, and 4469, 18.75%, 15.75% and 21.53% were modern contraceptives users for 2003, 2008 and 2014 respectively. The multiple cox proportional hazards model analysis identified place of residence and the educational level of a woman as strong predictors of modern contraceptives use in Ghana. Modern contraceptive use is increasing among rural residence. Women who are in formal occupations (professional, clerical, services) are more likely to use modern contraceptives than their colleagues in less formal occupations (manual, agricultural, sales).

**Conclusion:**

This study highlights the trends of determinants on modern contraceptive use in Ghana from 2003 to 2014. The most persistent determinants of modern contraceptive use in Ghana during this time period are place of residence and a woman’s educational level. Women working in Agriculture and Sales are the least users of modern contraceptives in Ghana over the period.

## Background

Globally, modern contraceptive use is fast increasing (63%) but continues to be low in Sub- Saharan Africa [1]. The latest 2014 Demographic and Housing Survey (DHS) in Ghana reports that the prevalence of modern contraceptive use among women of reproductive ages is 22%. Family planning has been identified as the key measure to help nations achieve the Sustainable Development Goal five (SDG-5) which aims at achieving gender equality and empowering all women and girls [2]. A number of researches have been done in Ghana and other countries to identify factors associated with the low uptake of modern contraceptives. Their findings indicate that contraceptives use is the cause of the high fertility rates in Sub-Saharan African countries resulting in early childbearing, high infant mortality and many other negative effects on the socio-economic situation in a country. [3]. According to the GDHS 2014, the use of modern contraceptive is low among women aged 15–19 (19%) and women age 45–49 (18%) [4]. In a study done in Vietnam the researcher describes the relationship between women’s age and modern contraceptive methods as an inverted ‘U-shape’. While the likelihood of contraceptive use was low among women aged 15–24, it was lower among the those 35 and above and highest among women aged 25–35 [5].

The educational level of a woman can also influence her acceptance and use of modern contraceptive methods. A study conducted in Bangladesh on prevalence and determinants of contraceptive use among employed and unemployed women revealed that employed women with higher educational levels had a marked increased probability of contraceptive use compared to illiterates [6]. A study done in Nigeria by Igbodkwe (2014), reveals that women with higher (tertiary) education were four times more likely to use modern contraceptives compared to those with lower educational level attainment [7]. In the same vein, women whose husbands have attained higher educational statuses were more likely to accept and approve the use of modern contraceptives methods [8, 9].

Type of residence has also been found by many researchers to be significantly associated with the use of modern contraceptive methods. Even though the majority of people reside in rural areas, women in the urban areas have higher odds of using modern contraceptive methods than women in rural areas. [7, 10].

Wealth index and type of earnings of a woman determines her income status as well as her affordability and accessibility status in connection with modern contraceptives [11, 12]. Marital status of a woman can influence her acceptance and use of a modern contraceptive [5, 7, 13]. Cultural factors, religion and source of information have all links to the beliefs of the women and have been identified to have an influence on choice and use of modern contraceptive methods [11, 14–17].

The purpose of this analysis is to assess the trends and determinants of the use of modern contraceptives among Ghanaian women of reproductive age. It is also to determine whether clustering has an influence on the use of modern contraceptives in Ghana. Clustering is considered in this study in order to access and control for heterogeneity if found to be present between participants from different areas or localities with different characteristics. Due to the multi-stage (complex) nature of the survey, there is a high probability of overestimation of the standard errors if all participants are treated as if they come from the same locality and with similar characteristics. This study seeks to determine and identify consistent determinants and others of modern contraceptive use from 2003 to 2014 among the currently non-pregnant married Ghanaian women population. The most influencing and consistent determinants that will be identified based on these findings will serve as priority intervention areas that the Ghana Ministry of Health and other health partners can concentrate on to improve on the use of modern contraceptives in Ghana.

## Methods

### Data source

The secondary data were obtained from the Ghana Demographic and Household Surveys from 2003 to 2014 through the DHS programme data access portal. These nationally representative surveys have all studied participants representatively selected from the ten (10) regions of Ghana, stratified according to urban and rural areas. A review of all the survey reports state that a two stage sampling design was used for all the selected periods. Based on this technique, the first stage has to do with the selection of Enumeration Areas (EA), referred to in this paper as clusters, from an updated master sampling frame constructed from previous surveys. This is usually done using systematic sampling with a probability proportional to the population size and number of households within the cluster. This is then followed by the listing of all households within the selected clusters to provide a sample frame for the second stage. In the second stage, households are systematically selected from all the clusters to provide adequate estimates for key indicators with acceptable precision. The variables for this analysis were extracted from the 2003, 2008, and 2014 Ghana Demographic and Health Survey’s (GDHS) structured questionnaires. The inclusion criteria were women who slept in the selected households the night before the day of the interview. The total number of eligible women interviewed in 2003, 2008, and 2014 were 5691, 4916, and 9396 respectively. In our analysis (a complete case analysis), only participants who provided responses to all variables (dependent – contraceptive use and independent – religion, education, age, wealth index and others) were used. All error entries and missing values for at least an observation or a variable were dropped. The sample sizes that was finally used for the analysis were: 2229, 2356, and 4469 respectively for 2003, 2008, and 2014. Written informed consent was obtained during the data collection process by the DHS for all participants within the ages of 15 to 49, details can be found at Ghana Statistical Service [4].

### Study outcome

Modern contraceptive use among currently married non-pregnant women of reproductive ages 15–49 years is the outcome variable as was capture in the DHS. Women within the ages 15–49 years were asked if they used any contraceptive to delay or avoid conception. Those who responded yes were further asked of the type of contraceptive method they used. The different types of fertility control methods used in this analysis were categorized into two: traditional methods referred to as non-use (rhythm, withdrawal, and folk methods) and modern contraceptives methods (pills, female and male sterilization, IUD, injectable, implants, male and female condom, diaphragm, and emergency contraception, LAM) according to Ghana Statistical Service [4].

### Statistical analysis

The analysis focused on the use of modern contraceptives, and all the analyses were done using STATA/IC version 14.1. The chi-squared test statistics was used to determine whether there was a statistical significant difference among women who use modern contraceptives over the years. In order to select and include variables in our multivariable analysis, all the variables were assessed at the bivariate level. Variables were therefore included after having showed some significance at this level of analysis. The simple Cox proportional hazards model was used for this purpose. Statistical significance of explanatory variables were considered under an alpha-level of 0.05 with a confidence level of 95%. Even though the data sets were obtained by cross sectional surveys, a Cox proportional hazards model was used for the analysis. This, because it has been showed by Breslow that by imposing a condition of constant follow up time, the Cox’s model can be adapted for the estimation of prevalence rate ratios in cross sectional studies (British Journal of Industrial Medicine 1993; 50:861–864). It is therefore a better alternative to logistic regression when equal times of follow-ups are assigned to all individuals. In order to ensure that the variance of the coefficients was not overestimated to result in wider confidence intervals compared to those in the binomial distribution (logistic regression), a robust variance estimator was used. Hence a Cox regression model with the same follow up time for each participant via a robust variance estimator was implemented. The hierarchical nature of the data demanded that a multilevel regression model be used in order to obtain a more accurate and reliable coefficient estimate of the model parameters and their standard errors. Modelling at the household level did not show any significant association with modern contraceptive use, it was therefore omitted from the entire model, but cluster was adjusted for in accounting for unobserved variations that may exist between clusters where individuals are nested. Cluster was considered as a level-2 variable while that of individual observations was assigned level-1. In the absence of any significant unobserved cluster effect, this model reduces to an ordinary Cox proportional hazards model.

## Results

The total number of observations identified based on the variables of interest over the period was 9054. The percentages of modern contraceptives use in Ghana was 21.53% in 2014, 15.75% in 2008 and 18.75% in 2003. There was an increase in modern contraceptives use in 2014 compared to 2003 and 2008. In the analysis, there was a significant difference (an association between users and study year) among modern contraceptive users over the fifteen-year period with a chi-square value of 33.68 and a corresponding *p*-value < 0.001. Among the women that used modern contraceptives, rural residence accounted for 66.9% in 2003 and reduced to 64.2% in 2008 and further dropped to 56.30% in 2014. The results also showed that there is low (< 25%) use of modern contraceptives among both rural and urban residence for all the periods (2003, 2008 & 2014). The majority of the women had no formal education, this increased from 35.27% in 2008 to 36.30% in 2014. By wealth index ratings, the majority of the respondents are always in the poorest category. These formed 27.37% of all respondents in 2003, slightly increased to 27.50% and further increased to 30.23% in 2014. Christians formed the majority of the respondents with most of the Christian women belonging to charismatic denominations, their number increased from 49.7% in 2008 to 54.92% in 2014. A lot (47.51%, 48.94% and 46.95% respectively for 2003, 2008, and 2014) of the women have children between one (1) and three (3). In 2003 81.78% of married women did not use modern contraceptives, this increased to 84.94% in 2008, and decreased to 78.59% in 2014 compared to their colleges who are just living with partners.

Results of the hierarchical modelling for both the crude and adjusted estimates are contained in Table 1. The results show that place of residence is statistically significantly associated with modern contraceptive use with prevalence rate ratios (PRR) for 2003 to be PRR: 0.63 at 95% CI (056, 0.81); 0.77 for 2008 and 95% CI (0.63, 0.94) and 1.21 at 95% CI (1.05, 1.39). After adjusting for all other variables, using the 2014 data, women who reside in the rural areas were 5% more likely to use modern contraceptives compared to urban women.

**Table 1:** A Hierarchical Cox Proportional Hazards Model Analysis of Modern Contraceptive use for 2003, 2008 & 2014

Predictive Variables	2003			2008			2014			
	Crude PRR	U_01_	Adjusted PRR	Crude PRR	U_01_	Adjusted PRR	Crude PRR	U_01_	Adjusted PRR	U_12_
Res Occupation	0.99(0.95, 1.05)	0.039		1.00(0.95, 1.06)	0.053		1.01(0.98, 1.04)	0.093		0.271
Manual	Reference	0.01	1	Reference	0.038	1	Reference		1	
Professional	1.33(0.85, 2.10)		1.09(0.59, 2.03)	1.58(0.98, 2.56)		2.21(1.27, 3.86)**	1.02(0.66, 1.35)		1.23(0.84, 1.87)	
Clerical	1.31(0.62, 2.77)		1.22(0.55, 2.71)	1.16(0.47, 2.85)		1.34(0.54, 3.3)	0.13(0.66, 1.92)		1.69(0.99, 2.91)	
Sales	1.05(0.81, 1.36)		0.94(0.72, 1.21)	0.94(0.68, 1.31)		0.86(0.61, 1.21)	0.92(0.78, 1.08)		0.96(0.82, 1.14)	
Agriculture	0.73(0.56, 0.95)*		0.85(0.63, 1.16)	0.87(062, 1.21)		1.09(0.75, 1.58)	0.88(0.74, 1.05)		0.89(0.75, 1.07)	
Services	1.01(0.68, 1.48)		0.89(0.60 1.32)	1.17(0.79, 1.73)		1.08(0.72, 1.62)	1.04(0.68, 1.59)		1.13(0.75, 1.70)	
Age	0.96(0.91, 1.01)	0.04		0.95(0.89, 1.00)	0.06		0.89(0.87, 0.93)***	0.097		
45–49	Reference	0.029	1	Reference	0.05	1	reference	0.094	1	
15–19	0.88(0.31, 2.53)		1.73(0.59, 5.06)	0.75(0.26, 2.19)		1.28(0.42, 3.89)	0.96(0.49, 1.89)		1.41(0.72, 2.78)	
20–24	2.08(1.23, 3.51)**		3.33(1.92, 5.77)***	2.16(1.35, 3.45)**		3.02(1.82, 5.01)***	2.02(1.51, 2.71)***		2.63(1.93, 3.59)***	
25–29	2.47(1.49, 4.11)***		3.32(1.96, 5.60)***	1.69(1.06, 2.68)*		2.23(1.36, 3.65)**	1.98(1.56, 2.51)***		2.31(1.79, 2.97)***	
30–34	2.44(1.49, 3.96)***		3.32(1.79, 4.81)***	2.02(1.28, 3.20)**		2.26(1.41, 3.62)**	1.88(1.48, 2.39)***		1.94(1.51, 2.49)***	
35–39	2.42(1.46, 3.99)**		2.93(1.58, 4.23)***	2.17(1.39, 3.36)**		2.29(1.49, 3.52)***	1.52(1.17, 1.97)**		1.49(1.13, 1.95)***	
40–44	2.29(1.37, 3.87)**		2.21(1.32, 3.70)**	1.58(0.97, 2.56)		1.65(1.03, 2.66)*	1.48(1.12, 1.95)**		1.44(1.09, 1.90)**	
Place of Residence	0.02(0.56, 0.81)***	0.007		0.77(0.63, 0.94)*	0.039		1.21(1.05, 1.39)**	0.088		
Urban	Reference		1	Reference		1	Reference		1	
Rural	0.02(0.56, 0.81)***		0.79(0.60, 1.03)	0.77(0.63, 0.94)*	0.039	0.90(0.68, 1.19)	1.21(1.05, 1.39)**		1.05(0.87, 1.25)	
Number__of Children	1.08(0.98, 1.19)	0.042		1.04(0.93, 1.16)	0.052		1.05(0.98.1.13)	0.923		
No Child	Reference		1	Reference		1	Reference		1	
One to Three	1.67(0.99, 2.81)		1.95(1.19, 3.19)**	1.54(0.95, 2.49)		1.84(1.14, 2.98)**	2.49(1.39, 4.47)**		2.59(1.44, 4.69)**	
Four to Six	1.97(1.17, 3.71)*		3.34(2.01, 5.55)***	1.68(1.02, 2.76)		2.73(1.59, 4.69)***	2.85(1.58, 5.14)**		3.94(2.16, 7.20)***	
Seven to Nine	1.88(1.06, 3.33)*		4.65(2.59, 8.32)***	1.57(0.81, 2.75)		3.53(1.84, 6.74)***	2.17(1.18, 3.99)*		4.07(2.16, 7.69)***	
Ten and Above	0.87(0.29, 2.54)	0.035	3.15(1.09, 9.08)*	0.69(0.21, 2.25)	0.052	1.95(0.58, 6.61)	1.06(0.43, 2.61)		2.19(0.89, 5.38)	
Educational Level	1.33(1.20, 1.47)***			1.20(1.08, 1.34)**	0.028		1.12(1.05, 1.20)***	0.904		
No Education	Reference		1	reference		1	Reference		1	
Primary	1.58(1.19, 2.08)**		1.24(0.92, 1.68)	1.36(1.03, 1.78)*		1.22(0.81, 1.84)	1.47(1.24, 1.73)***		1.27(1.08, 1.51)**	
Secondary	1.85(1.47, 2.31)***		1.32(0.98, 1.78)	1.55(1.21, 1.98)***		1.43(1.07, 1.91)*	1.36(1.17, 1.59)***		1.32(1.11, 1.57)**	
Higher	2.13(1.26, 3.59)**		1.55(0.74, 3.27)	1.33(0.74, 2.39)	0.027	0.81(0.37, 1.76)	1.27(0.93, 1.74)	0.795	1.48(0.94, 2.32)	
Wealth Index	1.16(1.09, 1.24)***			1.14(1.06, 1.22)***	0.024		0.96(0.91, 1.00)	0.091		
Poor	Reference		1	Reference		1	Reference		1	
Poorest	1.72(1.23, 2.39)**		1.31(0.94, 1.82)	1.03(0.75, 1.41)		0.97(0.69, 1.36)	1.11(0.93, 1.34)		0.99(0.83, 1.17)	
Middle	1.63(1.16, 0.29)**		1.12(0.77, 1.63)	1.31(0.94, 1.81)		1.27(0.85, 1.89)	1.08(0.89, 1.31)		0.91(0.74, 1.12)	
Richer	1.77(1.28, 2.45)**		1.07(0.69, 1.63)	1.65(1.21, 2.26)**		1.72(1.10, 2.68)*	1.07(0.88, 1.29)	0.079	0.92(0.72, 1.17)	
Richest	2.14(1.21, 1.48)***		1.16(0.71, 1.89)	1.51(1.08, 2.09)*	0.023	1.69(1.03, 2.80)*	0.75(0.59, 0.93)*		0.65(0.48, 0.87)	
Partner Educational Level	1.33(1.21, 1.48)***			1.91(1.07, 1.32)**	0.032		1.01(0.99, 1.03)	0.089		
No education	Reference		1	Reference		1	Reference		1	
Primary	1.92(1.33, 2.76)***		1.50(1.04, 2.18)	1.32(0.89, 1.95)		1.22(0.81, 1.84)	1.48(1.20, 1.82)***		1.21(0.99, 1.48)	
Secondary	2.03(1.54, 3.22)***		1.39(1.01, 1.89)	1.49(1.16, 1.96)		1.27(0.91, 1.76)	1.31(1.10, 11.55)**		1.18(0.99, 1.39)	
Higher	2.23(1.54, 3.22)***		1.40(0.89, 2.21)	1.58(1.10, 2.27)	0.032	1.15(0.74, 1.77)	1.09(0.86, 1.38)	0.764	1.02(0.78, 1.35)	
Partner Occupation	1.02(0.98, 1.06)	0.351	0.98(0.95, 1.02)	0.97(0.92, 1.01)	0.051	0.96(0.92, 1.00)	1.01(0.99, 1.03)	0.093	0.99(0.97, 1.02)	
Type of Earnings	0.81(0.71, 0.92)*	0.02	0.96(0.83, 1.12)	1.12(0.98, 1.29)	0.043	1.17(1.01, 1.36)	0.95(0.89, 1.01)	0.088	0.95(0.87, 1.04)	
Religion	0.99(0.95, 1.48)	0.039		0.93(0.89, 0.96)***	0.007		0.95(0.92, 0.98)**	0.078		
No religion	Reference		1	reference		1	reference		1	
Orthodox	2.48(1.29, 4.79)		1.63(0.82, 3.27)	1.61(0.94, 2.75)		1.42(0.83, 2.43)	1.26(0.88, 1.81)		1.15(0.79, 1.66)	
Charismatic	1.70(0.84, 3.44)		1.63(0.82, 3.24)	1.40(0.82, 2.41)		1.23(0.71, 2.14)	1.31(0.94, 1.83)		1.23(0.87, 1.73)	
Islam	0.70(0.25, 1.91)		1.34(0.65, 2.73)	1.01(0.55,1.85)		0.85(0.46, 1.59)	0.95(0.65, 1.39)		0.90(0.61, 1.33)	
Traditional	0.92(0.87, 0.97)**		0.79(0.29, 2.15)	0.83(0.39, 1.72)	0.008	0.82(0.39, 1.73)	0.59(0.33, 1.09)	0.175	0.58(0.32, 1.04)	
Ethnicity	0.92(0.87, 1.65)		1.04(0.97, 1.11)	0.99(0.94, 1.04)	0.05	1.13(1.06, 1.20)***	0.98(0.95, 1.02)	0.091	1.02(0.99, 1.06)	
Knowledge of Ovulation Cycle	0.97(0.92, 1.01)	0.036	0.98(0.94, 1.03)	0.97(0.93, 1.02)	0.05	1.00(0.96, 1.05)	0.98(0.94, 1.01)	0.934	0.99(0.96, 1.04)	
From Radio	1.69(1.30, 2.21)***	0.002		1.16(0.95, 1.43)	0.045		1.08(0.95, 1.22)	0.094		
No	Reference		1	Reference		1	Reference		1	
Yes	1.69(1.30, 2.21)***		1.32(1.01, 1.74)*	1.16(0.95, 1.43)	0.045	1.00(0.80, 1.25)	1.08(0.95, 1.22)		0.04(0.91, 1.019)	
From TV	1.42(1.18, 1.71)***			1.32(1.08, 1.62)**	0.038		1.03(0.90, 1.16)	0.094		
No	Reference		1	Reference		1	Reference		1	
Yes	1.42(1.18, 1.71)***		0.96(0.76, 1.23)	1.32(1.08, 1.62)**	0.038	1.10(0.87, 1.40)	1.03(0.90, 1.16)		1.00(0.86, 1.16)	
Newspapers	1.42(1.15, 1.77)**			0.83(0.56, 1.23)	0.054		0.79(1.05, 1.11)	0.093		
No	Reference		1	Reference		1	Reference		1	
Yes	1.42(1.15, 1.77)**		1.05(0.82, 1.34)	0.83(0.56, 1.23)	0.054	0.58(0.38, 0.88)	0.79(0.57, 1.11)		0.77(0.54, 1.09)	
FP Worker	1.09(0.87, 1.39)	0.04		1.42(1.09, 1.84)**	0.045		1.23(1.05, 1.45)*	0.087		
No	Reference		1	Reference		1	Reference		1	
Yes	1.09(0.87, 1.39)		1.08(0.86, 1.36)	1.42(1.09, 1.84)**	0.045	1.44(1.11, 1.86)**	1.23(1.05, 1.45)*		1.61(0.98, 1.37)	
Marital Status	1.26(0.94, 1.69)	0.032		1.23(0.99, 1.53)	0.047		1.02(0.88, 1.19)	0.093		
Married	reference		1	Reference		1	Reference		1	
Living together	1.26(0.94, 1.69)		1.33(1.00, 1.77)	1.23(0.99, 1.53)	0.047	1.20(0.95, 1.52)	1.02(0.88, 1.19)		0.95(0.81, 1.10)	
Desire for Children	0.77(0.66, 1.0.91)**	0.035		0.97(0.94, 1.01)	0.052		0.97(0.94, 0.99)**	0.099		
Both Same	Reference		1	Reference		1	Reference		1	
Hus. More	0.64(0.50, 0.82)***		0.75(0.59, 0.95)	0.96(0.74,1.25)		0.95(0.72, 1.24)	0.80(0.69, 0.92)**		0.87(0.76, 1.00)	
Hus. Fewer	0.77(0.56, 1.08)		0.70(0.49, 0.99)	1.25(0.86, 1.81)		1.12(0.78, 1.61)	0.91(0.74, 1.11)		0.94(0.77, 1.15)	
Don’t Know	0	0.024		0.83(0.64, 1.08)	0.044	0.90(0.69, 1.17)	0.77(0.65, 0.92)**	0.0942	0.87(0.73, 1.03)	
Decision on Respondent’s Health	1.03(0.97, 1.09)	0.044		0.96(0.89, 1.04)	0.053		0.95(0.89, 1.01)	0.882		
Res. Alone	Reference		1	Reference		1	Reference		1	
Respondent and Someone	1.25(0.97, 1.59)		1.13(0.88, 146)	1.25(0.98, 1.61)		1.24(0.98, 1.58)	1.39(1.19, 1.61)***		1.25(1.08, 1.45)**	
Husband Alone	1.08(0.87, 1.32)		0.09(0.97, 1.49)	0.98(0.74, 1.29)		0.98(0.74, 1.31)	0.91(0.73, 1.13)		0.89(0.73, 1.11)	
Someone Else	1.44(1.00, 2.08)	0.041	1.41(0.98, 2.03)	0.89(0.14, 5.63)	0.046	1.09(0.17, 7.21)	0.97(0.34, 2.76)	0.662	0.99(0.34, 2.85)	

*PRR* Prevalence rate ratio, *FP worker* Family planning worker, *From TV* From television, *Res* Respondent, *Hus* Husband, U_01_: cluster variation for each variable, U_12_: overall cluster variation for all variables, p-value < 0.05 = *, p-value < 0.01 = ** and p-value < 0.001 = ***

The age of a woman was not significantly associated with the use of modern contraceptives at the unadjusted level of analysis for 2003 (PRR: 0.96; 95% CI: 0.91, 1.01) and 2008 (PRR: 0.95; 95% CI: 0.89, 1.00). However, the prevalence rate ratio recorded in 2014 (PRR: 0.89; 95% CI: 0.87, 0.93) though showing ‘less likely use’ was highly statistically significant at *p* < 0.001. Adjusting for all the predictive variables under study, it is noticed that there is a positive influence on the association between age and modern contraceptive use, with a positive increase. Women in the age brackets of 15–19 and 45–49 years are the least users of modern contraceptives compared to all the other age categories. In 2003 women within the 15–19 year group were 73% more likely to use modern contraceptives compared to women within the 45–49 year groups. This decreased to 28% in 2008 and positively increased with 41% more likely to use modern contraceptives among women in the same age category in 2014. In 2014, women within the age groupings 30–34, 35–39 and 40–44 observed a decrease in the rate ratios of modern contraceptives use compared to what was recorded in the immediate pass survey (2008), observing a 94%, 49% and 44% more likely use of modern contraceptives respectively.

Women with 1–3 children in the unadjusted analysis showed a 67% more likely use of modern contraceptives compared to women without children in 2003; and this decreased to 54% in 2008. The 2014 analysis, however, saw a twofold more likely use of modern contraceptives among this group of women, with a significant statistical association (*p* < 0.01). After adjusting for all the other predictive variables in 2003, there was a 95% more likely use of modern contraceptives among women with children 1–3. This decreased to 84% more likely use in 2008 and in 2014 a twofold likely use of modern contraceptives was observed among this category of women - all referenced to women without children. It was also noticed in the adjusted analysis that women with ten children and above were 3.15 times more likely to use of modern contraceptives compared to women without children. It decreased to 95% more likely use of modern contraceptives among this category of women compared to women without children in 2008, and increased positively in 2014 by observing a twofold use of modern contraceptives among women with ten children and above, compared to women without children. Educational level of women at the unadjusted level of analysis showed high statistical association with modern contraceptive use in 2003 (PRR: 1.33, at 95% CI: 1.20, 1.47)***, 2008 (PRR: 1.20, at 95% CI: 1.08, 1.34**), and 2014 (PRR: 1.12, at 95% CI: 1.05, 1.20***). The adjusted analysis revealed no statistical association as was the case with the crude analysis. The adjusted analysis for 2003 showed that women with a higher educational attainment were 55%, secondary education 32% and primary education level 24% s were more likely to use modern contraceptives compared to women with no formal education. In 2014, the adjusted analysis revealed that women with a higher educational l attainment level were 48% more likely to use modern contraceptives than those with secondary education (32%) and primary education (27%) compared to women with no formal education.

The wealth index was found to be associated with modern contraceptive use in 2003 (PRR: 1.16 at 95% CI: 1.09, 1.24) ***, and 2008 (PRR: 1.14, at 95% CI: 1.06, 1.22) ***, with the exception of 2014 (PRR: 0.96, at 95% CI: 0.91, 1.00) in which year it was not. The adjusted analysis for 2003 showed that women from the poorest households were 31% more likely to use modern contraceptives. Women from richest, middle, and richer households were 16%, 12%, and 7% more likely to use modern contraceptives - all referenced to women from poorer households. In 2008 the crude analysis showed that women from richer (PRR: 1.65, at 95% CI: 1.21, 2.26)** households were more likely to use modern contraceptives, than women from poorest (3%), middle (31%) and richest (51%) households, when compared to women categorized poorer households. The adjusted analysis also showed a 72% increased use of modern contraceptives among women from richer households, with statistical significance; this is an increase over what was recorded in the same category in 2003. In 2014, the unadjusted analysis revealed that the higher the household wealth index, the lower the likely use of modern contraceptives. Women from richest households were 25% (PRR: 0.75, at 95% CI: 0.59, 0.93)* less likely to use modern contraceptives compared to women from poorer wealth index households.

Religion, as one of the independent predictors, was associated with modern contraceptive use in 2008 (PRR: 0.93, at 95% CI: 0.89, 0.96)*** and 2014 (PRR: 0.95, at 95% CI: 0.92, 0.98)**. The adjusted analysis in 2003 however revealed that modern contraceptive use among women belonging to either orthodox and charismatic denominations were equal (63%), with Islamic women recording a 34% more likely use of modern contraceptives as against women with no religious faith. In 2008, the adjusted analysis showed a general reduction in the use of modern contraceptives in all the religious denominations. Women within the orthodox churches recorded a 42% more likely use of modern contraceptives, and the charismatics recorded a 23% more likely use of modern contraceptives. Muslim women were 15% less likely to use modern contraceptives than women with no religious faith. In 2014, orthodox denominations were 15% more likely to use modern contraceptives, this was a reduction from what was recorded in both 2003 and 2008. Women within charismatic denominations recorded a 23% more likely use of modern contraceptives, a reduction in what was noticed in 2003 and 2008 with reference to women with no religious faith. Even though women belonging to the Islamic faith were 10% less likely to use modern contraceptives compared to women with no religion, it was still an improvement over what was recorded in 2008.

The cluster variable was included in the dataset and controlled for and found to be significant for some of the variables under study in the bivariate as well as the multivariable analyses for all the periods. It was therefore necessary to account for the heterogeneity of participants’ location in order to generate accurate standard errors estimates. More specifically, in 2014 a cumulative cluster level variance of 0.271 was recorded after adjusting for all covariates under study. Details of the other variables can be found in Table 1.

## Discussion

The results of this analysis revealed the prevalence of modern contraceptive use in Ghana as 18.75% in 2003, 15.75% in 2008 and 21.53% in 2014. The prevalence as observed in this study is similar to that reported in the Ghana Demographic and Health Survey study, where 2003 recorded 18.7%, 2008 recorded 16.6% and that for 2014 was 22.2%. The prevalence of this study showed a statistically significant difference over the period determined via a chi-squared test statistic. The results of this analysis revealed in the final adjusted model for all the periods under study that the higher the education level of a woman, the higher her likelihood to use modern contraceptives. In 2014, women who had primary education as the highest level of education attainment were 27% more likely to use modern contraceptives than women who had no formal education. Women who attained higher educational levels were 48% more likely to use modern contraceptives than women without formal education. This finding, though significant, is lower than what was observed by Balew et al. which showed that women with higher education have a six fold higher odds of FP acceptance than those with no education [18]. The 2014 results further showed that the higher a woman’s wealth index, the less her likelihood of using modern contraceptives. This is seen in the adjusted analysis, where the results showed that women from the poorest households were 1% less likely to use modern contraceptives. Women from the richest households were 35% less likely to use modern contraceptives than women from poorer homes. This is in line with a study in Nigeria which revealed that there was 74% less likelihood of contraceptive use among women from richest homes compared to a 70% less likely use of contraceptives among women from poorest households [2]. A similar study done in Ghana revealed that women with high wealth status are less likely to use contraceptives than women with low wealth status [3]. Place of residence can to a large extent, by default, influence the type of work a person will most probably be doing for a living. It was found that the use of modern contraceptives is increasing among rural residents e in Ghana. In 2003, adjusting for all the factors under study, 22% of rural resident women were less likely to use modern contraceptives compared to urban resident women. This decreased to 10% less likely use in 2008, but increased to 21% more likely use of modern contraceptives among rural resident women in 2014 compared to urban resident women. This is in line with a study done in Ethiopia by Worku et al. [19], which reported that modern contraceptive use increased from 2.4% among rural residence to 11.9% in 2005 and further increased to 28.9% in 2011 [19]. This improvement in Ghana can be attributed to the priority given to the Community- based Health Planning Services (CHPS) concept by the government of Ghana in collaboration with the Ministry of Health. The Ghana Health Service and other Reproductive Health NGOs have brought health care services to the door steps of rural residents that are affordable to families and individuals. A study in Uganda revealed that there is a downward trend in rural – urban variations in modern contraceptive use, reporting an odds of 73% lower rate in 1995. The 2003 and 2008 results of this study are also in line with the results of a study conducted in the Asuogyaman District of Ghana [20]. The study concluded that the use of modern contraceptives was higher in urban areas than in the rural areas [20]. A woman’s type of earning determines her economic independence and her contribution to the household expenses. This, to a large extent, empowers her in partaking in major family decisions, including in the use of contraceptives. The findings for all the periods revealed that women who are paid in cash compared to women who are not paid at all, use more modern contraceptives than women paid only in kind as well as women paid both in cash and kind. This is contrary to what was noticed in a study on inequality in the fertility rate and the use of modern contraceptives among Ghanaian women from 1988 to 2008. That study revealed that the non-use of modern contraceptives was 66.5% or 3% more likely among women with low income than among women with high income (53.2%) [7]. The findings of this study are also in line with the findings of a study on trends of modern contraceptive use among young married women, based on the 2000, 2005, and 2011 Ethiopian Demographic and Health Surveys [19]. The results revealed a higher use of modern contraceptives among women who are paid in cash for working than among women who are not working and women who are working but not paid [19]. The results of this study also revealed that the use of modern contraceptives is lower among women with less than three children for all the periods. It was constantly high among women with between four and nine children.

Modern contraceptive use is higher among Christians than among Muslims. There was a sharp decrease in modern contraceptives use among orthodox Christians between 2008 and 2014, the use of modern contraceptives is, however, higher among charismatic Christians than among orthodox Christians. The findings of this study are supported by other studies. The results of these findings contradict those of a study conducted in Mozambique which showed a higher prevalence of modern contraceptive use among Catholics than among traditional Protestants [21].

In the unadjusted analyses for all the periods, it was observed that the very strong and consistent predictors of modern contraceptive use among women of reproductive ages in Ghana are: place of residence and a woman’s level of education, after accounting for cluster variation. Presented in either two of the three age periods, strong predictors of modern contraceptive use among women of reproductive ages in Ghana are wealth index, partner, educational level, television as source of information, health education by FP workers, and desire for children. The results of these findings are in line with the findings of a research done on predictors of modern contraceptive use in Ethiopia, which identified among other predictors, place of residence, possession of radio, and income, as significant predictors of modern contraceptives use [22]. The results of this study contradict the findings of Tsehaye et al. [23] as the results of their findings identified place of residence, ethnic groups, religion and income as not associated with modern contraceptives [23]. However, the educational level of women, marital status and age were identified to by associated with modern contraceptives use [23]. This study also revealed that clustering have an effect on modern contraceptive use in Ghana, though it varies from year to year and from variable to variable. This can be attributed to the empirical reasons that individuals within specific clusters will most likely share similar influencing factors and characteristics. The results of these findings are in line with what was identified in a study on Family Planning Promotion, Contraceptive Use and Fertility Decline in Ghana that noticed a cluster level variance of 0.15. That means that there is considerable variations in contraceptive use rates between clusters [11].

### Study strength and weakness

The main strength of this study, is the analytic method used, that is, the multilevel or mixed effects Cox proportional hazards model.. This regression model was used due to its advantage over the logistic model to determine associations (prevalence rate ratios) between the dependent (dichotomous) and the independent variables. The representativeness of the participants was guaranteed because the survey was conducted by both national and international experts within the area. The analysis done with the Ghana Demographic Health Survey stopped at just the univariate level. This study went beyond just only that by applying both bivariate and multivariable approaches. An important limitation of this study is that strong conclusions could not be drawn with respect to the causes of modern contraceptive use. This is due to the cross-sectional design of the survey, hence causality could not be established.

## Conclusion

The trend analysis of the 2003, 2008, and 2014 GDHS data sets revealed a high use of modern contraceptives among urban resident women. However, there is a gradual increase in the use of modern contraceptives among rural women. The study also found higher use of modern contraceptives among Christian women than among Muslim women, even though it was noticed that there is a gradual increase in use among Muslim women. Some of the factors associated with modern contraceptive use in Ghana are: place of residence and the educational status of a woman. Others include wealth index, partner, educational level, television as source of information, health education by FP workers, and desire for children. Others include: age of the woman, type of earnings, religion, ethnicity, radio as source of information and newspapers/magazines as sources of information. In order to achieve a higher prevalence of contraceptive use, more education is needed especially targeting Muslim women. Family planning workers should be encouraged and motivated to help educate the women. To reach a number of women, sources such television, radio and magazines should be used as a tool to reach the targeted group. Education on the use of contraceptives should not target only women but also the women partners due to partner’s influence on their usage. More local authorities and men groups need to be targeted. Establishment of community health committees to help educate the people will also be in the right direction. To achieve these, the Ghana Health Service with the support of other organizations need to make resources available to the Regional Health Directorates for them to also distribute same to the District Health Directorates. This must be done with an effective monitory and evaluation plan strictly implemented from National to the District levels.

## Abbreviations

CHPS: Community based health post services
CI: Confidence interval
DHS: Demographic health survey
EA: Enumeration areas
FP: Family planning
GDHS: Ghana demographic health survey
HR: Hazard ratio
IUD: Intrauterine device
LAM: Lactation amenorrhea
NGO’s: Non-governmental organizations
SDG - 5: Sustainable development goal five
